# Prevalence of Foot Pain Across an International Consortium of Population‐Based Cohorts

**DOI:** 10.1002/acr.23829

**Published:** 2019-04-25

**Authors:** Lucy S. Gates, Nigel K. Arden, Marian T. Hannan, Edward Roddy, Tiffany K. Gill, Catherine L. Hill, Alyssa B. Dufour, Trishna Rathod‐Mistry, Martin J. Thomas, Hylton B. Menz, Catherine J. Bowen, Yvonne M. Golightly

**Affiliations:** ^1^ University of Southampton, Southampton, and University of Oxford Oxford UK; ^2^ University of Southampton, Southampton, UK, University of Oxford, Oxford, UK, and University of Sydney Sydney Australia; ^3^ Harvard Medical School Boston Massachusetts; ^4^ Keele University and Haywood Hospital Staffordshire UK; ^5^ University of Adelaide Adelaide South Australia Australia; ^6^ University of Adelaide, Adelaide and The Queen Elizabeth Hospital Woodville South Australia Australia; ^7^ Keele University Staffordshire UK; ^8^ La Trobe University Melbourne Victoria Australia; ^9^ University of Southampton Southampton UK; ^10^ University of North Carolina Chapel Hill

## Abstract

**Objective:**

Despite the potential burden of foot pain, some of the most fundamental epidemiologic questions surrounding the foot remain poorly explored. The prevalence of foot pain has proven to be difficult to compare across existing studies due to variations in case definitions. The objective of this study was to investigate the prevalence of foot pain in several international population‐based cohorts using original data and to explore differences in the case definitions used.

**Methods:**

Foot pain variables were examined in 5 cohorts: the Chingford 1000 Women Study, the Johnston County Osteoarthritis Project, the Framingham Foot Study, the Clinical Assessment Study of the Foot, and the North West Adelaide Health Study. One question about foot pain was chosen from each cohort based on its similarity to the American College of Rheumatology pain question.

**Results:**

The precise definition of foot pain varied between the cohorts. The prevalence of foot pain ranged from 13% to 36% and was lowest in the cohort in which the case definition specific to pain was used, compared to the 4 remaining cohorts in which a definition included components of pain, aching, or stiffness. Foot pain was generally more prevalent in women and obese individuals and generally increased with age, with the prevalence being much lower in younger participants (ages 20–44 years).

**Conclusion:**

Foot pain is common and is associated with female sex, older age, and obesity. Estimates of the prevalence of foot pain are likely to be affected by the case definition used. Therefore, in future population studies, the use of consistent measures of data collection must be considered.

## Introduction

Foot pain has been identified as an independent risk factor for locomotor disability 1, impaired balance 2, increased risk of falls 3, 4, loss of independence, and reduced quality of life 5. It is likely that foot pain contributes a significant burden in both older individuals and healthcare systems. The literature suggests that foot pain is highly prevalent in the general population; however, prevalence estimates vary from 9% to 30% 6, 7, 8, 9. Foot problems have been reported to account for up to 8% of a general practitioner's consultations for musculoskeletal disorders in the UK 10, 11.

**Significance & Innovations**

- Comparison of original data is a key component to effectively enhance the scientific content and value of large studies, both past and current. This study represents the first effort to compare original data for foot pain, which is an understudied yet common concern in rheumatology.
- As seen with previous data harmonization of knee osteoarthritis‐related outcomes, the prevalence of foot pain is likely affected by the case definition used.
- Rather than using summary estimates of effect in future work, the use of original participant data across cohorts allows for a more detailed consideration of the heterogeneity in variable case definitions.
- In future population studies, use of more consistent measures of data collection must be considered.

Despite the potential burden of foot pain, to date, some of the most fundamental epidemiologic questions surrounding the foot remain poorly explored, particularly with consideration to basic demographic features. Accurately estimating the burden of foot pain in the general population is important so that clinical and cost‐effective management strategies can be implemented. Estimating the proportion of a population with a condition such as foot pain will provide the basis for determining the number of people who may require care, for monitoring changes in the condition occurrence over time. An investigation of foot pain prevalence using original data in a number of international population‐based cohorts would enable determination of the differences in foot pain frequency between geographic regions and sociodemographic groups, with consideration of age, sex, body mass index (BMI), and race. Frequencies obtained from research are the basis for probability estimates for the purposes of patient care, and future research can begin to establish potential risk factors for foot pain and associated conditions.

Traditional meta‐analyses can be valuable and efficient in terms of time and resources required but can be affected by several substantial limitations. They are limited to published results and may therefore be subject to publication bias, and the quality and availability of data may vary across studies 12. Such issues have been previously encountered due to the considerable variation used in case definitions for type, period, and patterns of pain, which limited the ability to pool data and provide accurate prevalence estimates 7. The heterogeneity of variable case definitions is a limitation to any research aimed at comparing data across cohorts or study data sets. It is necessary to identify the components and definitions of each variable and, when possible, to produce a method to standardize each variable. Such methods have been previously highlighted in the investigation of knee osteoarthritis (OA) 13, 14.

Therefore, the primary aim of this study was to identify the prevalence of foot pain in 5 prospective cohorts, using original participant data. The secondary aim was to consider potential reasons for differences in pain across geographic locations according to important factors such as age, sex, BMI, race, selection bias in each cohort (sampling method, response rate, and loss to follow‐up), and measurement bias (foot pain case definitions). This cross‐sectional study makes use of original data from 5 international population cohorts linked to an international consortium of foot and ankle research collaborators.

## Patients and Methods

### Cohort selection

Early findings from a cross‐cohort foot OA collaboration project with principal investigators from prospective cohorts, including the Chingford 1000 Women Study, the Johnston County Osteoarthritis Project, and the Framingham Foot Study, revealed a need to establish a larger consortium of foot and ankle research collaborators in order to address variations in data collection across population cohorts. In 2017, a consortium of international foot and ankle research collaborators was formed to encourage a more collaborative approach to research on foot and ankle pain. The consortium consisted of principal investigators and researchers associated with current foot and ankle cohort studies and representative research. Potential cohorts for the current study were identified through members of the consortium with knowledge of prospective population‐based cohorts rich in foot pain data. The Chingford 1000 Women Study 15, the Johnston County Osteoarthritis Project 16, the Clinical Assessment Study of the Foot 17, the Framingham Foot Study 18, and the North West Adelaide Health Study were identified 19. The characteristics of participants in each cohort are shown in Table 1.

**Table 1:** Demographic characteristics of each cohort^a^

	Chingford 1000 Women Study	Johnston County Osteoarthritis Project	Framingham Foot Study	Clinical Assessment Study of the Foot	North West Adelaide Health Study
Data collection time point	Year 15 (2003)	First follow‐up visit (1999–2004)	Phase 1 (2002 and 2008)	Baseline health survey (2010–2011	Stage 2 clinic (2004–2006)
No. of participants (at time point)	655	1,619	3,420	4,490	3,145
Age, mean ± SD years	68.6 ± 5.8	65.8 ± 9.8	66.5 ± 10.6	64.9 ± 9.8	47.6 ± 17.5
Age group, years					
20–34	–	–	–	–	889 (28.3)
35–44	–	–	17 (0.5)	–	644 (20.5)
45–54	–	203 (12.5)	451 (13.2)	741 (16.5)	577 (17.7)
55–64	206 (31.5)	592 (36.6)	1,208 (35.3)	1,624 (36.2)	428 (13.6)
65–74	308 (47.0)	484 (29.9)	944 (27.6)	1,334 (29.7)	320 (10.2)
≥75	141 (21.5)	340 (21.0)	800 (23.4)	791 (17.6)	307 (9.8)
Sex					
Male	–	581 (35.9)	1,449 (43.8)	2,198 (49.0)	1,545 (49.1)
Female	655 (100)	1,038 (64.1)	1,921 (56.2)	2,292 (51.0)	1,600 (50.9)
BMI, mean ± SD kg/m^2^	27.2 ± 4.8	30.2 ± 6.3	28.4 ± 5.5	27.5 ± 5.2	27.8 ± 5.7
BMI category, kg/m^2^					
<18.5	10 (1.5)	13 (0.8)	23 (0.7)	62 (1.4)	43 (1.4)
18.5–24.9	228 (34.8)	290 (17.9)	937 (27.4)	1,480 (33.0)	1,014 (32.3)
25.0–29.9	241 (36.8)	588 (36.3)	1,335 (39.0)	1,808 (40.3)	1,169 (37.2)
≥30.0	176 (26.9)	728 (45.0)	1,125 (32.9)	1,140 (25.4)	919 (29.2)
Race					
White	655 (100)	1,158 (71.5)	3,420 (100)	4,395 (97.9)	–
African American	–	461 (28.5)	–	–	–
African Caribbean	–	–	–	14 (0.3)	–
Asian	–	–	–	49 (1.1)	–
African	–	–	–	8 (0.2)	–
Other	–	–	–	24 (0.5)	–

a Except where indicated otherwise, values are the number (%). BMI = body mass index.

### Sampling methods and data collection in the cohort populations

#### Chingford 1000 Women Study

The Chingford 1000 Women Study is an ongoing prospective, population‐based longitudinal study that was established to assess risk factors and associations with osteoporosis and OA 15. The cohort originally consisted of 1,003 women ages 45–64 years who were recruited from a general practice in Chingford, North East London, UK. Since 1989, the women have been assessed almost annually with a number of investigations. The current study used data from year 15 (2003).

#### Johnston County Osteoarthritis Project

The Johnston County Osteoarthritis Project is an ongoing population‐based longitudinal study that was established to investigate the epidemiology of OA among both African American and white individuals residing in 6 townships in a mostly rural county in North Carolina 16. Individuals who were recruited for this study were civilian, noninstitutionalized residents who were at least 45 years old. The original cohort included women enrolled between 1991 and 1997. Data for the current analysis were from the first follow‐up visit and were collected from 1999 to 2004.

### Clinical Assessment Study of the Foot

The Clinical Assessment Study of the Foot is an ongoing population‐based, prospective, observational cohort study of foot pain and foot OA 17. All adults ages 50 years and older who were registered with 4 general practices in North Staffordshire, UK, were invited to take part in the study, irrespective of consultation for foot pain or problems. In the current study, we used data from the initial baseline health survey questionnaire mailed in 2010/2011, which was used to gather information on aspects of general health, including foot pain.

#### Framingham Foot Study

The Framingham Foot Study includes members of the Framingham Heart Study Original Cohort, the Framingham Heart Study Offspring Cohort, and a third community sample 18. The Original Cohort was formed in 1948 from a two‐thirds sample of the town of Framingham, Massachusetts, in order to study risk factors for heart disease and has been examined biennially 20. In 1972, the Offspring Cohort (comprised of offspring and spouses of the offspring) was formed in order to study familial risk factors for heart disease; the cohort has been examined every 4 years 21. The community sample was derived from census‐based random digit dialing within the Framingham community; subjects who were older than age 50 years and ambulatory were contacted in order to increase participation by minorities. Data for the current analysis were collected between 2002 and 2008.

#### North West Adelaide Health Study

The North West Adelaide Health Study is a longitudinal study of randomly selected adults who were ages 18 years and older at the time of recruitment (1999 to 2003) from the northwest region of Adelaide, South Australia. The aim of this study is to increase the ability of strategies and policies to prevent, detect, and manage a range of chronic conditions 19. Participant information was obtained from a computer‐assisted telephone interview (CATI), a self‐completed questionnaire, and a clinic assessment at each stage 19, 22. In the current study, data collected during stage 2 (2004–2006) were used.

### Inclusion criteria

Across all included cohorts, participants who had responded to the foot pain question were selected for analysis. When available, information regarding age, sex, BMI, and race was also extracted for each participant.

### Statistical analysis

Descriptive data for demographic characteristics of each cohort are presented as the mean ± SD or frequencies and percentages, as appropriate. Prevalence and 95% confidence intervals (95% CIs) were also calculated for foot pain according to age, sex, BMI, and race for each cohort. Sensitivity analysis of the Chingford 1000 Women Study was undertaken to estimate the prevalence of foot pain, with adjusted cutoff points (6+/15+ days).

The Chingford 1000 Women Study and Johnston County Osteoarthritis Project data analyses were undertaken at Oxford University, using Stata version 14.1. The remaining cohort analyses were undertaken in‐house; Clinical Assessment Study of the Foot using Stata version 14; Framingham Foot Study using SAS version 9.4; North West Adelaide Health Study using SPSS version 24 and Stata version 14.2.

### Ethics approval

The Chingford 1000 Women Study was approved by the Outer North East London Research Ethics Committee, and written consent was obtained from each woman. The Johnston County Osteoarthritis Project was approved by the institutional review boards at the University of North Carolina and the Centers for Disease Control and Prevention. The Clinical Assessment Study of the Foot was approved by the Coventry research ethics committee (REC reference 10/H1210/5), and written consent was obtained from all participants. The Framingham Foot Study was approved by the Hebrew SeniorLife and Boston University Medical Center institutional review boards, and participants provided written, informed consent prior to enrollment. The North West Adelaide Health Study was approved by the Human Research Ethics Committee of The Queen Elizabeth Hospital, Adelaide, South Australia. All participants provided written informed consent.

## Results

### Response rates and loss to follow‐up

#### Chingford 1000 Women Study

Of the original cohort of 1,003 participants, 658 (65.6%) returned at year 15 (in 2003) and completed a joint symptom questionnaire. Three of these participants (0.6%) were excluded from the current study due to missing data for foot pain, leaving 655 participants for analysis.

#### Johnston County Osteoarthritis Project

Of the original cohort of 3,187 participants, 1,739 (54.6%) returned for the first follow‐up clinic visit (1999 to 2004). One hundred twenty (6.9%) of these participants were excluded from the current study due to missing data for either demographics or foot pain, leaving 1,619 participants for analysis.

#### Clinical Assessment Study of the Foot

The baseline health survey questionnaire was mailed to 9,334 adults and completed by 5,109 (adjusted response 56%). Of these, 619 individuals (12.1%) were excluded from the current study due to missing data for either the foot pain questions or demographics, leaving 4,490 participants for analysis.

#### Framingham Foot Study

A total of 3,429 individuals were included in the baseline data collection between 2002 and 2008. Nine of these individuals (0.3%) were excluded from the current study due to missing data for either foot pain questions or demographics, leaving 3,420 participants for analysis.

#### North West Adelaide Health Study

A total of 4,056 individuals comprised the original cohort; 3,205 of these individuals (79.0% of the eligible sample) participated in all 3 data collections (CATI survey, self‐completed questionnaires, and clinical assessments) during stage 2, between 2004 and 2006. Of these, 60 subjects (1.9% of the stage 2 sample) were excluded due to missing data for either foot pain or demographics, leaving 3,145 subjects for analysis.

### Standardization of foot pain

Each cohort was examined for available questions about foot pain. In each cohort, the foot pain questions were assessed for differences in the duration of pain (i.e., any/most days) and the period of recall (i.e., in the last month/last year/ever). Because there was a variation in pain duration and recall between a number of the questions in the cohorts, one question about foot pain was selected from each cohort based on its similarity to the question: “Have you had pain (in either foot) on most days in the last month?” 13 (Table 2).

**Table 2:** Harmonization of foot pain variables across cohorts^a^

Cohort	Original question	Responses standardized to match “pain on most days”
Chingford 1000 Women Study	“On how many days in the last month did you get pain?” (0/1–5/6–14/15+ days)	Pain in either foot on most days (L/R)Pain on most days (yes) = pain on at least 15 daysPain on most days (no) = pain on fewer than 15 days
Johnston County Osteoarthritis Project	“On most days do you have pain, aching or stiffness in your feet?” (yes/no)	Pain in either foot on most days (L/R)YesNo
Framingham Foot Study	“On most days do you have pain, aching or stiffness in your feet?” (yes/no)	Pain in either foot on most days (L/R)YesNo
Clinical Assessment Study of the Foot	“Pain, aching or stiffness in the foot in the past month” (no days/few days/some days/most days/all days)	Pain in either foot on most days (L/R)Pain on most days (yes) = most days/all days and had foot pain in the last yearPain on most days (no) = no days/few days/some days and had foot pain in the last year OR did not have foot pain in the last year
North West Adelaide Health Study	“Pain, aching or stiffness in the foot in the past month” (no days/few days/some days/most days/all days)	Pain in either foot on most days (L/R)YesNo

a In each cohort, the foot pain questions were assessed for differences in the duration of pain and the period of recall.

**Figure 1:**
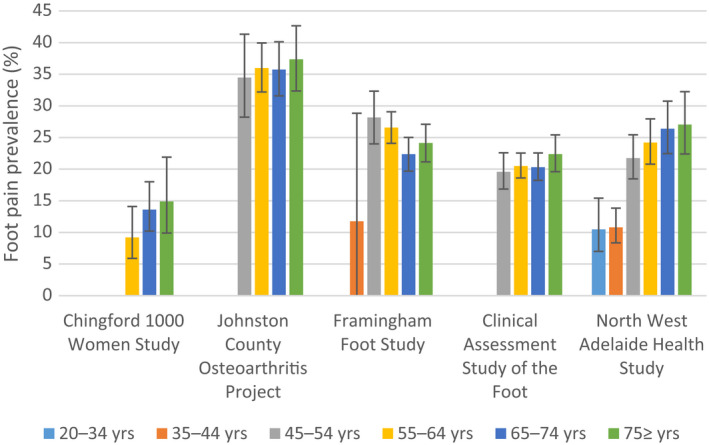
Prevalence of foot pain across cohorts according to age group. Vertical lines with error bars represent the 95% confidence intervals.

**Figure 2:**
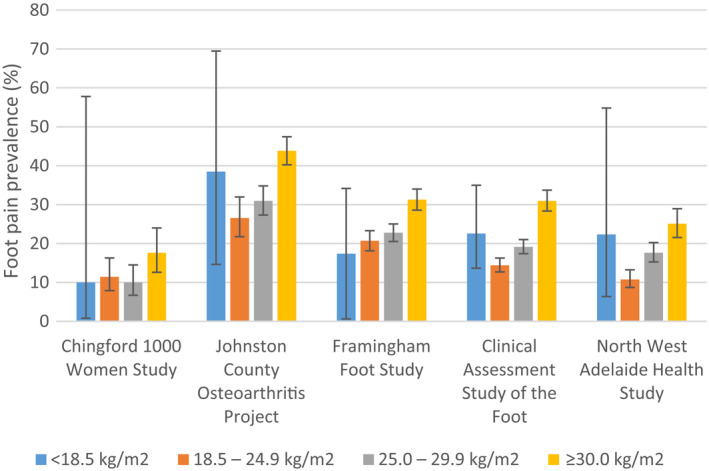
Prevalence of foot pain across cohorts according to body mass index category. Vertical lines with error bars represent the 95% confidence intervals.

The prevalence of foot pain ranged from 13% to 36% between cohorts. Table 3 shows the prevalence of foot pain in each cohort, stratified by age, sex, BMI, and race. Foot pain was more prevalent in women than in men across all cohorts in which data for both sexes were available. The largest absolute difference between men and women in the occurrence of foot pain was 11% in the Framingham Foot Study. Prevalence ranged from 9% to 36% in participants ages 55–64 years, 14% to 36% in those ages 65–74 years, and 15% to 37% in those ages 75 years and older (Figure 1). In all cohorts, foot pain was most prevalent in participants classified as obese (BMI >30.0 kg/m^2^) (Figure 2). In the Johnston County Osteoarthritis Project, the Clinical Assessment Study of the Foot, and the North West Adelaide Health Study, the prevalence of foot pain was also high in those with a BMI lower than 18.5 kg/m^2^; however, the numbers of participants were small, and the 95% CIs were wide. Race was reported in 4 cohorts, 2 of which were limited to only white participants (Chingford 1000 Women Study and Framingham Foot Study). The prevalence of foot pain among white participants ranged from 13% to 36%. In the Johnston County Osteoarthritis Project, the frequency of foot pain was comparable in white individuals and African Americans (36% and 35%, respectively). When data for other races were available within the Clinical Assessment Study of the Foot, the prevalence of foot pain was highest in African Americans (38% compared to only 10% in Asian participants); however, the numbers of these participants were small, and the 95% CIs were wide.

**Table 3:** Prevalence of foot pain in the cohorts, stratified by age, sex, body mass index (BMI), and race^a^

	Chingford 1000 Women Study (n = 655)	Johnston County Osteoarthritis Project (n = 1,619)	Framingham Foot Study (n = 3,420)	Clinical Assessment Study of the Foot (n = 4,490)	North West Adelaide Health Study (n = 555)
Foot pain	12.5(10.2–15.3)	36.0(33.7–38.4)	25.0(23.5–26.4)	20.6(19.5–21.8)	17.7(16.0–19.4)
Age group, years					
20–34	–	–	–	–	10.5(7.0‐15.4)
35–44	–	–	11.8(0.0–28.8)	–	10.8(8.4–13.8)
45–54	–	34.5(28.2–41.3)	28.2(24.0–32.3)	19.6(16.0–22.6)	21.8(18.5–25.4)
55–64	9.2(5.9–14.1)	36.0(32.2–39.9)	26.6(24.1–29.1)	20.5(18.6–22.5)	24.2(20.8–28.0)
65–74	13.6(10.2–18.0)	35.7(31.6–40.1)	22.4(19.7–25.0)	20.3(18.2–22.6)	26.4(22.5–30.8)
≥75	14.9(9.9–21.9)	37.432.4–42.7)	24.1(21.2–27.1)	22.4(19.6–25.4)	27.022.4–32.2)
Sex					
Male	–	30.5(26.9–34.3)	19.0(17.0–21.0)	18.3(16.7–20.0)	15.3(13.2–17.7)
Female	12.5(10.2–15.3)	39.1(36.2–42.1)	29.6(27.6–31.7)	22.9(21.2–24.6)	19.9(17.5–22.5)
BMI category (kg/m^2^)					
<18.5	10.0(0.8–57.8)	38.5(14.6–69.5)	17.4(0.6–34.2)	22.6(13.7–35.0)	22.3(6.4–54.8)
18.5–24.9	11.4(7.9–16.3)	26.6(21.8–32.0)	20.7(18.1–23.3)	14.4(12.7–16.3)	10.8(8.7–13.2)
25.0–29.9	10.0(6.7–14.5)	31.0(27.3–34.8)	22.8(20.5–25.0)	19.1(17.4–21.0)	17.6(15.3–20.2)
≥30.0	17.6(12.6–24.0)	43.8(40.2–47.5)	31.3(28.6–34.0)	31.0(28.3–33.7)	25.1(21.6–29.0)
Race					
White	12.5(10.2–15.3)	36.4(33.7–39.3)	25.0(23.5–26.4)	20.8(19.6–22.0)	–
African American	–	34.9(30.7–39.4)	–	–	–
African Caribbean	–	–	–	21.4(6.0–54.0)	–
Asian	–	–	–	10.2(4.2–22.9)	–
African	–	–	–	37.5(8.7–79.2)	–
Other	–	–	–	12.5(3.7–34.5)	–

a Values are the percent (95% confidence interval). BMI = body mass index.

## Discussion

This is the first study to use original data to compare the prevalence of foot pain across multiple international populations. The prevalence of foot pain ranged from 13% in the Chingford 1000 Women Study to 18% in the North West Adelaide Health Study, 21% in the Clinical Assessment Study of the Foot, 25% in the Framingham Foot Study, and 36% in the Johnston County Osteoarthritis Project. The study highlights the differences in foot pain across age, sex, BMI, and race while considering differences in the case definitions used for variables, which is a vital consideration when combining or comparing data across multiple data sets.

In cohorts that included both men and women, the prevalence of foot pain was consistently higher in women. This difference has been widely reported 6, 7, 9, 23, with a suggested partial attribution to lifetime footwear habits, although other factors such as occupation and family history are also thought to contribute 18, 24. Women are more likely than men to report musculoskeletal pain in general, and consideration should also be given to sex‐related variations in pain perception 25, hormonal influences 26, and psychological and social factors 27. However, the role of other potential sex differences such as occupation or physical activity levels is currently unknown. The overall prevalence of foot pain was actually lowest in the Chingford 1000 Women Study, which included only women. While unknown factors such as comorbidities may play a role, this is likely due to the case definition used for foot pain. In the Chingford 1000 Women Study, the question was specific to pain only, in comparison to all other cohorts in which questions included pain, aching, and stiffness. This difference challenges whether the use of questions including aching and stiffness may overestimate pain. The original foot pain question in the Chingford 1000 Women Study allowed for a categorical response of 0, 1–5, 6–14, and 15+ days. For the purposes of standardization with the remaining 4 cohorts in this study, all of which used a foot pain duration of “most days,” a cutoff of 15+ days was chosen to represent most days in the Chingford 1000 Women Study. This cutoff point was identical to that used in a previous study to represent painful knee OA 28. However, because no explicit number of days was provided to participants in the Chingford 1000 Women Study to represent “most” days, it cannot be assumed that all participants would classify 15+ days as most days. A sensitivity analysis was therefore undertaken to estimate the prevalence of foot pain, with an adjusted cutoff point of 6+ days, to capture participants who answered 6–14 days. The prevalence of foot pain increased from 12.5% (15+ days) to 18% (6+ days), thus highlighting the sensitivity in prevalence estimates according to the question response components.

The prevalence of foot pain generally increased with age and was much lower in younger participants (ages 20–44 years) compared to those older than age 45 years. This increase is consistent with that observed in previous studies (7, 23). Although differences in foot pain prevalence can be seen in each decade above the age of 45 years, overlapping 95% CIs suggest there is little difference in these prevalence estimates. Results of a systematic review and a survey study showed a stronger positive association of foot pain with age among women compared with men 7, 9. This may in part be due to sex differences in pain perception, because women are known to report more severe levels of pain, more frequent pain, and pain of longer duration compared to men 25, 27. In addition, the higher frequency of pain‐related conditions such as OA is seen more commonly in women and older persons (29).

In all cohorts, the prevalence of foot pain was highest in participants classified as obese. Foot pain was more prevalent at the lower and upper extremes of the BMI in the Johnston County Osteoarthritis Project, the Clinical Assessment Study of the Foot, and the North West Adelaide Health Study; however, small numbers of participants and wide 95% CIs in the low BMI category (<18.5 kg/m^2^) suggest that these estimates should be interpreted with caution. The prevalence of foot pain increased incrementally with increasing BMI in the Framingham Foot Study. Previous cross‐sectional studies have also demonstrated associations between increasing BMI and foot pain 31, 32, in particular fat mass (30, 32). There is also evidence from longitudinal studies that BMI is a predictor of incident foot pain over 5 years (33), and fat mass is a predictor of incident foot pain over 3 years (34).

Race data were largely limited to the white demographic, with foot pain prevalence lower in both UK cohorts than in the US cohorts. In the bi‐racial cohort of the Johnston County Osteoarthritis Project, the occurrence of foot pain was similar between whites and African Americans. Within the Clinical Assessment Study of the Foot, the prevalence of foot pain was similar between African Caribbeans and whites. It was highest in Africans and lowest in Asians and others; however, interpretation of these findings is limited, because only 2% of the sample were racial/ethnic minorities (non‐white). Previous studies also showed significant racial/ethnic differences in the prevalence of common foot disorders, independent of sex or education. Two previous studies using data not included in the current study also demonstrated differences between races. In the Feet First study, the total number of foot conditions such as toe deformities, flat feet, corns, calluses, skin pathologies, and ankle joint pain were found to be more prevalent in African Americans than in non‐Hispanic whites and Puerto Ricans (35). In the Women's Health and Aging Study, significant differences in pain severity were found between races, with more foot pain observed in African American than in non‐African American participants (36).

It has been suggested that the differences in health conditions between racial and ethnic groups could be attributable to different levels of access to healthcare, different rates of chronic conditions (such as diabetes mellitus, obesity, or vascular disease) possibly associated with foot ailments, early life experiences, or occupational patterns that differ among groups independent of education (35). Because ethnicity is the term given for the culture of people in a given geographic region, including but not limited to language, religion, and customs, it would be beneficial to consider the role of ethnicity in the investigation of pain and/or chronic conditions. Further work is required to determine the etiologic factors for such differences.

The greatest challenge when comparing data across population cohorts is the heterogeneity that exists across factors such as recruitment methods, data collection time points, and variable definitions. Even when comparable variable definitions are used, there is often further heterogeneity in the measures used to collect data and the parameters of each variable. The main limitation in the current study was the variation in questions used to determine the presence of foot pain, particularly the duration of pain and the question response components, as shown from the response categories for the original pain questions in the Chingford 1000 Women Study. The results of a recent study showed that the variation in wording in National Health and Nutrition Examination Survey (NHANES)–type pain questions can result in variation in the prevalence of pain between 41% and 75% 13. Although the NHANES‐type questions were designed to capture joint pain related to OA, we cannot confidently confirm the cause of foot pain in all participants.

Participants in the Chingford 1000 Women Study and the Framingham Foot Study are predominantly white; therefore, results cannot be generalized to other races. Similarly, the Chingford 1000 Women Study is a women‐only cohort. Country of birth, but not race, was collected in the North West Adelaide Health Study. Those born in Australia were asked whether they are aboriginal or Torres Strait Islander (ATSI); however, only 11 individuals identified as an ATSI in stage 2. Country of birth does not represent the race categories used in the remaining 4 cohorts. The North West Adelaide Health Study has a predominantly white sample; therefore, country of birth was not included in the analysis.

Johnston County, North Carolina, is a semirural area in the southern US that includes a greater proportion of lower‐income residents than that observed in the populations from which other cohorts in the current study were derived (37). Foot pain frequency estimates for the Johnston County Osteoarthritis Project may be higher than those for other cohorts, because lower socioeconomic status is associated with a greater prevalence of musculoskeletal pain in adults (38, 39). We do expect that the prevalence of foot pain is likely high in the US, given that the cohort from Framingham, Massachusetts, has the second highest foot pain prevalence across these cohorts. In addition, high BMI, which is also a factor associated with foot pain (33), is more common in the Johnston County Osteoarthritis Project than in other cohorts.

Year 15 follow‐up was chosen in the Chingford 1000 Women Study due to the availability of a foot pain question at this time point. The inability to use baseline data resulted in a smaller sample size than that at the original baseline. Those who did not attend the year 15 follow‐up tended to be older and have a higher BMI at baseline compared to year 15 attendees who were selected for this study. For the Clinical Assessment Study of the Foot, response to the baseline health questionnaire was lower than expected (56%). However, responders did not differ greatly from the original mailed population by age, sex, or general practice (40). In the Johnston County Osteoarthritis Project, generally persons who did not return for the first follow‐up visit tended to be older, less educated, and more likely to be male and African American. In the North West Adelaide Health Study, stage 2 data collection was used for foot pain, because this was the first time musculoskeletal questions were asked in the cohort. Participants who failed to provide information at stage 2 tended to be younger, and the number of men was slightly higher than the number of women.

The strengths of this study are that the results are based on data derived from population‐based prospective observational cohorts, therefore enhancing generalizability and reducing the chance of selection bias. This study analyzed original participant data and was therefore not limited to the publication bias inherent when analyzing previously published results. Although most studies within a standard meta‐analysis use a variety of definitions of outcomes, the current study was able to minimize this variation by choosing similar questions at selected time points. This approach can be expanded to other time points and for other variables to enable longitudinal individual participant data meta‐analysis to identify risk factors for foot pain and associated conditions. Although the wording of pain questions differed for 2 of the cohorts, all 5 cohorts used questions that were specific to self‐reported foot pain.

This study provides useful comparisons of foot pain between 5 population cohorts. The comparisons showed that irrespective of geographic location, the prevalence of foot pain is higher among persons who are obese and lower in younger participants (ages 20–44 years). Although the prevalence of foot pain was lower in the younger population, it is important to recognize that foot pain does occur in this age group, which may warrant further investigation and clinical attention. Between‐cohort data for race were limited; however, within‐cohort results showed foot pain was potentially more prevalent in African Americans. Foot pain was also more prevalent in women than in men.

This study also highlights variation between cohorts in the manner in which pain data are collected. A degree of the variation in prevalence between cohorts may, at least in part, be attributable to the sensitivity of different definitions of pain. In particular, it is important to consider the effect that including all the components of pain, aching, or stiffness in one question may have on estimating the prevalence of pain only. Future population studies should use more consistent measures of data collection, and the role of question response categories should not be underestimated. Agreement on a standardized set of key questions about foot pain and measures would be useful for future prospective data collection phases within existing and newly establishing cohorts.

## Author Contributions

All authors were involved in drafting the article or revising it critically for important intellectual content, and all authors approved the final version to be published. Dr. Gates had full access to all of the data in the study and takes responsibility for the integrity of the data and the accuracy of the data analysis.

### Study conception and design

Gates, Arden, Hannan, Roddy, Gill, Bowen, Golightly.

### Acquisition of data

Gates, Hannan, Gill, Hill, Golightly.

### Analysis and interpretation of data

Gates, Arden, Hannan, Gill, Hill, Dufour, Rathod‐Mistry, Thomas, Menz, Bowen, Golightly.
